# Enhanced detection and serotyping of foot‐and‐mouth disease virus serotype O, A, and Asia1 using a novel multiplex real‐time RT‐PCR

**DOI:** 10.1111/tbed.14603

**Published:** 2022-06-25

**Authors:** Da‐Rae Lim, Soyoon Ryoo, Hyeonjeong Kang, Su Hong Oh, Sang‐Ho Jang, BoKyu Kang, Hye‐Jin Park, Hyeonwoo Hwang, Jae‐Myung Kim, Choi‐Kyu Park, Sang‐Ho Cha

**Affiliations:** ^1^ Foot‐and‐Mouth‐Disease Research Division Animal and Plant Quarantine Agency Gimcheon‐si Gyeongsangbuk‐do Republic of Korea; ^2^ College of Veterinary Medicine & Animal Disease Intervention Center Kyungpook National University Daegu Republic of Korea; ^3^ MedianDiagnostics Inc. Sunhwan‐daero, Dongnae‐myeon Chuncheon‐si Gangwon‐do Republic of Korea; ^4^ Animal and Plant Quarantine Agency Gimcheon‐si Gyeongsangbuk‐do Republic of Korea

**Keywords:** detection, foot‐and‐mouth disease, foot‐and‐mouth disease virus, multiplex, real‐time reverse transcription‐polymerase chain reaction, serotyping

## Abstract

Rapid and accurate detection and serotyping of foot‐and‐mouth disease (FMD) virus (FMDV) is essential for implementing control policies against emergent FMD outbreaks. Current serotyping assays, such as VP1 reverse transcription‐polymerase chain reaction (RT‐PCR)/sequencing (VP1 RT‐PCR/sequencing) and antigen detection enzyme‐linked immunosorbent assay (ELISA), have problems with increasing serotyping failure of FMDVs from FMD outbreaks. This study was conducted to develop a multiplex real‐time RT‐PCR for specific detection and differential serotyping of FMDV serotype O, A, and Asia 1 directly from field clinical samples. Primers and probes were designed based on 571 VP1 coding region sequences originated from seven pools. Multiplex real‐time RT‐PCR using these primers and probes demonstrated serotype‐specific detection with enhanced sensitivity compared to VP1 RT‐PCR/sequencing for reference FMDV (n = 24). Complete serotyping conformity between the developed multiplex real‐time RT‐PCR and previous VP1 RT‐PCR/sequencing was demonstrated using FMDV field viruses (n = 113) prepared in cell culture. For FMDV field clinical samples (n = 55), the serotyping rates of multiplex real‐time RT‐PCR and VP1 RT‐PCR/sequencing were 92.7% (51/55) and 72.7% (40/55), respectively. Moreover, the developed multiplex real‐time RT‐PCR demonstrated improved FMDV detection (up to 33.3%) and serotyping (up to 67.7%) capabilities for saliva samples when compared with 3D real‐time RT‐PCR and VP1 RT‐PCR/sequencing, during 10 days of challenge infection with FMDV serotype O, A, and Asia 1. Collectively, this study suggests that the newly developed multiplex real‐time RT‐PCR assay may be useful for the detection and differential serotyping of FMDV serotype O, A, and Asia 1 in the field.

## 1 INTRODUCTION

Foot‐and‐mouth disease (FMD) is a highly contagious transboundary disease of domestic and wild cloven‐hoofed animals caused by the foot‐and‐mouth disease virus (FMDV). FMD outbreaks are associated with considerable economic losses due to their effect on trade and decreased animal productivity (World Organisation for Animal Health [OIE], 2019). FMDV belongs to the genus *Aphthovirus* of the family *Picornaviridae* and consists of seven genetically and immunologically distinct serotypes (O, A, Asia 1, C, Southern African Territories [SAT] 1, SAT 2, and SAT 3). These serotypes have multiple topotypes and genetic lineages (Knowles & Samuel, 2003).

Based on genetic and antigenic analyses, FMDVs throughout the world have been geographically divided into seven regional pools (OIE/FAO Reference Laboratory Network for Foot‐and‐Mouth Disease Annual Reports, 2008), among which serotypes O, A, and Asia 1 in pool 1 have been threatening the livestock industry in the Republic of Korea. Current molecular assays for FMDV detection are reverse transcription‐polymerase chain reaction (RT‐PCR) or 3D real‐time RT‐PCR, while those for FMDV serotyping use antigen detection ELISA or sequencing of the amplified products from RT‐PCR for the VP1 coding region according to the World Organization for Animal Health (OIE) manual (OIE, 2019). Both assays may require virus isolation using cell lines for serotyping, due to insufficient amounts of amplified products by VP1 RT‐PCR and low amounts of viral proteins for Ag ELISA in FMD clinical samples. Therefore, new rapid and sensitive methods are required to support better effective FMD control policies including vaccination during FMD outbreaks (Bachanek‐bankowska et al., 2016).

Because VP1 coding sequences vary greatly depending on the FMDV serotype, serotyping can be achieved directly by RT‐PCR of the serotype‐specific VP1 coding region without sequencing (Alexandersen et al., 2000; Callens & de Clerq 1997; Le et al., 2011; Vangrysperre & de Clercq, 1996) However, these serotype‐specific RT‐PCRs are troublesome and unsuitable for routine use because of their low sensitivity and specificity (Reid et al., 2001). Therefore, several real‐time RT‐PCR assays have been developed for single serotype‐specific detection (Jamal & Belsham, 2015; Reid et al., 2014). Nevertheless, their serotyping methods were designed to specifically detect serotype O, A, and Asia 1 FMD viruses circulating in the Middle East (Reid et al., 2014) or West Eurasia (Jamal & Belsham, 2015), and not proved for detection of genetically variable FMDVs under the same serotypes circulating in other pools, which may lead to failure or delay in making serotype‐specific preventive controls including emergent vaccination against unprecedented serotypes of FMD outbreaks.

Therefore, this study was conducted to develop a multiplex real‐time RT‐PCR for specific detection and differential serotyping of FMDV serotype O, A, and Asia 1 from field clinical samples during FMD outbreak with enhanced performance.

## 2 MATERIALS AND METHODS

### 2.1 Viruses and cell cultures

FMDV reference strains were propagated in fetal porcine kidney cell line (LFBK‐α_V_β_6_) cultures, as described in a previous study (LaRocco et al., 2013). Viral isolation from clinical samples of FMD outbreaks was attempted using the cell line. Tissue homogenates (20%) were prepared in Dulbecco's modified eagle medium (Corning, NY, USA) and inoculated into LFBK‐α_V_β_6_ cell lines prepared one day before inoculation. The cells and culture media were harvested and stored at −80°C until use when a 90% cytopathic effect (CPE) was observed.

Seneca valley virus (SVV) and swine vesicular disease virus (SVDV) were also included in the evaluation to differentiate FMDV from other viruses causing FMD‐like clinical signs. SVV and SVDV strains were grown in pig kidney cell (IBRS)‐2 cultures as previously described in the World Organization for Animal Health (OIE) manual (OIE, 2019).

Cells were cultured in T‐75 cell culture flasks (Corning∖), using Dulbecco's‐modified eagle medium (Corning) for LFBK‐α_V_β_6_ cells and minimum essential medium (Corning) for IBRS‐2 cells, both supplemented with 2% antibiotic‐antimycotic (Gibco, NYC, USA) and 5% fetal bovine serum (Corning). Each cell line was counted using a TC20 automated cell counter (Bio‐Rad, CA, USA), and the concentration normalized to a seeding density of 1.3 × 10^7^ cells/mL was incubated at 37°C and 5% CO_2_ incubator.

### 2.2 Extraction of viral genomes

Viral RNAs were extracted from FMDVs, SVV, and SVDV propagated in cells, and from FMD field outbreak clinical samples and pig experiments, using the Nextractor NX‐48S DNA/RNA extraction kit (Genolution, Seoul, Republic of Korea), according to the manufacturer's instructions. For the extraction of viral RNA from clinical samples, 20% tissue homogenates were prepared in Dulbecco's‐modified eagle medium (Corning), and the viral RNA samples were eluted in 100 μL of nuclease‐free water and stored at −80°C until being evaluated by multiplex real‐time RT‐PCR, 3D real‐time RT‐PCR (Callahan et al., 2002), and VP1 RT‐PCR/sequencing.

### 2.3 3D real‐time RT‐PCR

The 3D real‐time RT‐PCR assay for FMDV was performed with 3D gene‐specific primers and probes according to previously described OIE‐recommended methods (Callahan et al., 2002), which are currently in use for the molecular diagnosis of FMD at the Animal and Plant Quarantine Agency (APQA). The 3D real‐time RT‐PCR assay was carried out using an AccuPower FMDV Real‐time RT‐PCR MasterMix Kit (Bioneer, Daejeon, Korea) and a CFX96 Touch real‐time PCR detection system (Bio‐Rad). A 50‐μL reaction mixture containing 44 μL of FMDV master mix, 1 μL of internal positive control (IPC), and 5 μL of template RNA was prepared, according to the manufacturer's instructions. The IPC consists of a partial mouse gene, which is *Dvl‐1*. The real‐time RT‐PCR program included reverse transcription at 45°C for 30 min, initial denaturation at 95°C for 5 min, followed by 45 cycles at 95°C for 15 s and 55°C for 50 s, and a final extension at 72°C for 5 min. Real‐time fluorescence values of 6‐carboxyfluorescein (FAM)‐labeled probes were measured in ongoing reactions at the end of each annealing step. The results were interpreted by identifying the threshold cycle (*Ct*) value for reporter dye detection. The sample was considered negative if a fluorescence signal was not detected within 45 cycles.

### 2.4 VP1 RT‐PCR/sequencing

RT‐PCR for FMDV VP1 coding region sequencing was performed with a primer set (forward primer: 5’‐AGYGCYGGYAARGAYTTTGA‐3’, reverse primer: 5’‐CATGTCYTCYTGCAT CTGGTT‐3’) designed to anneal within the VP3 coding region (forward primer) and the 2B coding region (reverse primer) to amplify the VP1 full‐length coding region as shown in the previous study (Le et al, 2012). RT‐PCR was performed using a commercial one‐step RT‐PCR kit (Qiagen, Hilden, Germany). Each 25 μL reaction mixture containing 5 μL of 5× reaction buffer, 0.6 μM of each primer, 0.1 μL of dNTP, 1 μL of enzyme mix, and 8 μL of FMDV RNA as template was prepared according to the manufacturer's instructions. Amplification was carried out in a thermal cycler (Applied Biosystems, MA, USA) under the following conditions: reverse transcription at 50°C for 30 min, initial denaturation at 95°C for 15 min, followed by 35 cycles at 94°C for 30 s, 55°C for 30 s, and 72°C for 60 s; and a final extension at 72°C for 10 min. VP1 PCR products were sequenced by a commercial company (Bionics, Seoul, Republic of Korea) using the Sanger method.

### 2.5 Multiplex real‐time RT‐PCR for FMDV detection and serotyping

#### 2.5.1 Design of primers and probes

A total of 571 full‐length VP1 coding sequences classified into seven different serotypes of FMDVs of seven pools (pool 1, n = 410; pool 2, n = 40; pool 3, n = 60; pool 4, n = 27; pool 5, n = 8; pool 6, n = 10; pool 7, n = 16), were collected from GenBank of the National Center for Biotechnology Information or by sequencing clinical samples of FMD outbreaks in our laboratory. The VP1 coding region sequences were aligned using BioEdit (http://www.mbio.ncsu.edu/bioedit/bioedit) and analyzed collectively. Following the analyses, VP1 conserved regions were identified as targets for primers and probes design for each serotype. Several primer and probe sets were designed and evaluated in multiple possible combinations to detect the homologous FMDV serotype to select the best‐performing primers and probes. Bioinformatic analysis was conducted using Fast‐PCR software 6.0.04 (PrimerDigital Ltd., Finland) for all the primers and probes to verify their specificity. These in‐silico evaluations were based on all available sequences of FMDV obtained from GenBank and our laboratory. Through the in‐silico PCR results, each set of primers and probes (Table 1) for the multiplex real‐time RT‐PCR was proven to have high specificity for the current circulating FMDVs. All primers and probes were synthesized by Bionics.

**TABLE 1:** Sequence of primers and probes designed for serotyping of FMDV using multiplex real‐time RT‐PCR in this study

Serotype	Primer/probe	Sequence (5ʹ‐3ʹ) *	Genome position**	Amplicon(pb)
O	Forward	ACTACGGTGCYAT**Y**AARGC**N**AC	3674‐3695	148
	Reverse	GCTGTTTCAC**M**GG**Y**GCCACAATCT ACTGCTTTACAGGTGCCACTATTT AGAAGCTGTTTTGCGGG**Y**GCCAC AAAGTCTGTTTCAC**M**GGTGCCAC	3794‐3816 3793–3817 3799–3821 3799‐3821	
	Probe	HEX‐TCGGCCCTCTTCAT**V**CGGTA**M**AGC‐BHQ1 HEX‐TCGGCTCTCTTCAT**K**CGGTAGAGT‐BHQ1 HEX‐GTCTGGGACA**R**TACGTCTCAGCTC‐BHQ1	3711‐3734 3711–3734 3728‐3751	
A	Forward	GGGA**R**TCAGCAGACCC**Y**GTCAC GGGAGTCTGCAGACCC**R**GT**Y**AC	3279‐3300 3279–3300	154
	Reverse	GTCTGCATGAGGTC**D**ATGAC**R**T	3411‐3432	
	Probe	FAM‐ATGGACAGGTT**Y**GTGCAGATCAAGC‐BHQ1 FAM‐ GCTTCAT**M**ATGGACAG**R**TTTGTGAAA‐BHQ1 FAM‐TGTGTCTCACC**R**CCGTAGTT**Y**TCAAC‐BHQ1	3371‐3395 3363–3388 3307‐3333	
Asia 1	Forward	GTTCT**Y**GACAGGTT**Y**GTGAAACT	3368‐3390	181
	Reverse	TGGGCGC**R**CC**R**TTGGGCACC TGGG**Y**G**M**GCCGTTGGGCACC	3529‐3548 3529‐3548	
	Probe	TxR‐TGCGACGTACTACTT**Y**TC**R**GACCTGG‐BHQ2	3469‐3494	

* Bold text in primer and probe sequences represent degenerate bases: Y, C, or T; R, A, or G; N, A, C, G, or T; M, A or C; V, A, C, or G; K, G or T; D, A, G or T

** Genome position of primer‐ and probe‐binding sequences according to the complete genome sequence of the foot‐and‐mouth‐disease virus isolate (FMDV), O/SKR/JC/2014 (GenBank ID: KX162590.1), A/Pocheon/001/KOR/2010 (GenBank ID: KC588943.1), and As1/Shamir/89 (GenBank ID: JF739177.1)

#### 2.5.2 Real‐time RT‐PCR protocol

The multiplex real‐time RT‐PCR assay for detection and serotyping of FMDV was performed using serotype‐specific primers and probes according to the following procedure, using a CFX96 Touch real‐time PCR detection system (Bio‐Rad). The 25 μL reaction mixture contained 12.5 μL of 2× one‐step RT‐PCR buffer, 0.5 μL of PrimeScript RT enzyme mix, 0.1–0.5 μM of each primer and probe, and 5 μL of template RNA, was prepared according to the manufacturer's instructions of the one step PrimeScript RT‐PCR kit (Takara Bio Inc., Shiga, Japan). The real‐time RT‐PCR program included reverse transcription at 42°C for 5 min, initial denaturation at 95°C for 10 s, 40 cycles of PCR at 95°C for 5 s and 60°C for 30 s, and cooling at 4°C for 10 s. Real‐time fluorescence values of probes labeled with hexachlorofluorescein (HEX) for serotype O, FAM for serotype A, and sulforhodamine 101 acid chloride (TxR) for serotype Asia 1 were measured in ongoing reactions at the end of each annealing step. The results were interpreted by identifying the *Ct* values for the reporter dyes. The sample was considered negative if a fluorescence signal was not detected within 40 cycles.

#### 2.5.3 Specificity and sensitivity

Multiplex real‐time RT‐PCR was evaluated for the capability of serotype‐specific detection and limit of detection (LOD), using cloned P1 genes and cell‐propagated viruses of field isolates of three different serotypes. P1 sequences of FMDV O/Andong/KOR/2010, A/Pocheon/SKR/2010, and As1/Shamir/89 viruses were amplified and cloned into the pTOP TA V2 vector (Enzynomics, Daejeon, Korea). The recombinant plasmid DNA samples were linearized by digestion with EcoRI (TaKaRa Bio, Kusatsu, Japan), purified using the Expin CleanUP SV Kit (GeneAll Biotechnology, Seoul, Korea), and in vitro transcribed and removed with the RiboMAX Large‐Scale RNA Production System‐T7 (Promega, Fitchburg, Wisconsin, USA) according to the manufacturer's instructions. The concentrations of each RNA were determined by measuring the absorbance at 260 nm using a NanoDrop Lite (Thermo Fisher Scientific, Waltham, MA, USA). RNA copy number per microliter (copies/μL) was estimated based on the molecular weight of each transcript and the Avogadro number. The transcribed RNAs from single serotype or mixed serotypes were 10‐fold serially diluted from 10^0^ to 10^7^ copies/μL and tested for detection and serotyping using the multiplex real‐time RT‐PCR. In addition, viral RNA was extracted from each 10^0^ to 10^7^ TCID_50_/mL dilution of FMDV serotype O (O/Anseong/KOR/2019), A (A/Gimpo/KOR/2018), and Asia 1 (As1/Shamir/89) and evaluated using multiplex real‐time RT‐PCR, 3D real‐time RT‐PCR, and VP1 RT‐PCR/sequencing assays.

#### 2.5.4 Detection and serotyping performance evaluation

##### 2.5.4.1 Serotyping using field‐isolated viruses and clinical samples

Serotyping identification with the multiplex real‐time RT‐PCR and the VP1 RT‐PCR/sequencing was evaluated for field viruses (n = 113) isolated from LFBK‐α_V_β_6_ cell lines, and for field clinical samples (n = 55) collected from FMD Asian outbreaks including the Republic of Korea (n = 3), Bangladesh (n = 32), Laos (n = 15), Vietnam (n = 25), and Cambodia (n = 93). For the isolation of FMDV in cell culture, clinical samples (tissues, saliva, and nasal discharge) were homogenized and filtered with a 0.22 mm syringe filter. The filtrate was inoculated into the cells. After CPE was observed, the isolated viruses were passed twice in cell culture. Serotyping of all viruses isolated from cell culture and field clinical samples was conducted using VP1 RT‐PCR/sequencing and multiplex real‐time RT‐PCR.

##### 2.5.4.2 Detection and serotyping using clinical samples from pig experiment

The detection and serotyping performance of the 3D real‐time RT‐PCR, the VP1 RT‐PCR/sequencing, and the multiplex real‐time RT‐PCR was evaluated with saliva from infected pigs. Eight‐week‐old specific‐pathogen‐free miniature pigs (n = 12) were divided into four groups (three heads per group) and housed in the Animal Biosafety Level‐3 facility at the APQA. The pigs were negative for antibodies or antigens of swine influenza virus, porcine reproductive and respiratory syndrome virus, FMDV, and mycoplasma hyopneumoniae (MH). Three groups were infected with 100 μL (10^5^ TCID_50_/mL) of FMDV serotype O (O/Anseong/KOR/2019), A (A/Gimpo/KOR/2018), and Asia 1 (As1/Shamir/89) through footpads, and one group was maintained as a negative group. Saliva samples were collected for 10 days, daily, after being challenged and stored at −80°C until use. All experiments were conducted in accordance with the guidelines of the Institutional Animal Care and Use Committee of APQA.

## 3 RESULTS

### 3.1 Specificity and sensitivity of multiplex real‐time RT‐PCR

**FIGURE 1:**
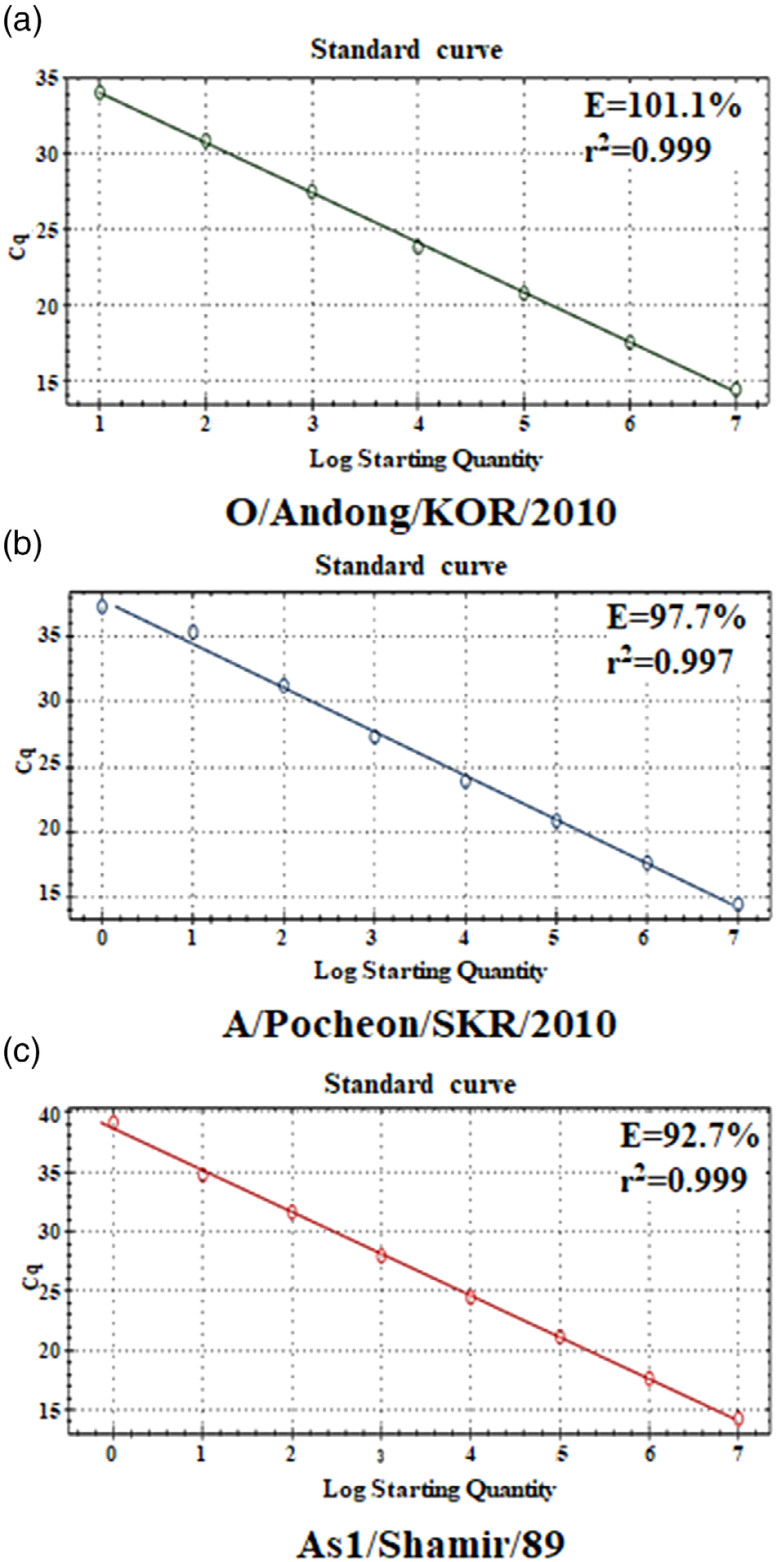
Standard curves of multiplex real‐time RT‐PCR using 10‐fold serial dilutions (10^0^–10^7^ copies/μL) of specific FMDV RNAs transcribed from P1 genes of O/Andong/KOR/2010 (a), A/Pocheon/SKR/2010 (b), and As1/Shamir/89 (c)

**FIGURE 2:**
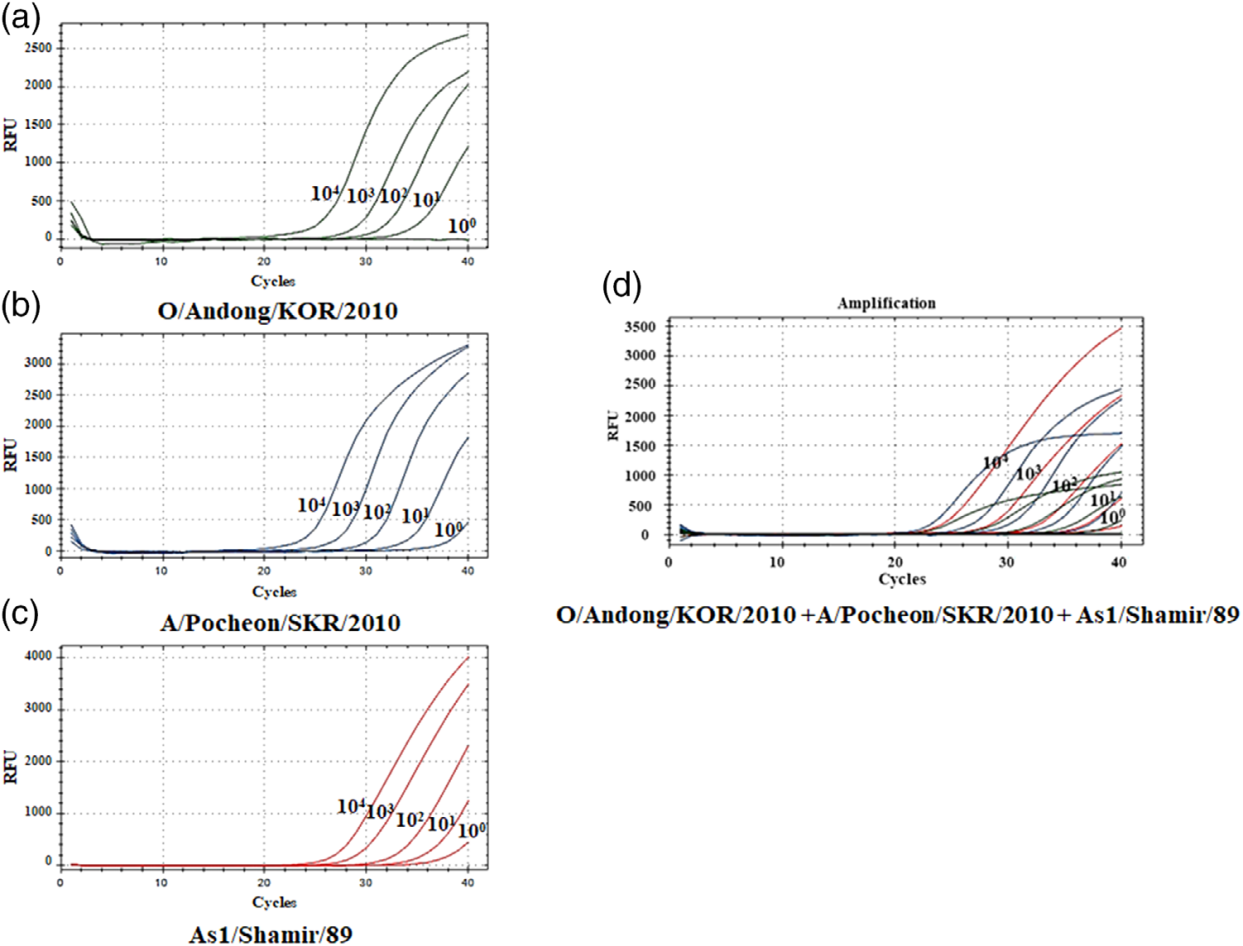
Amplification curve of multiplex real‐time RT‐PCR using 10‐fold serial dilutions (10^0^–10^7^ copies/μL) of transcribed FMDV RNAs transcribed from P1 genes of O/Andong/KOR/2010 (a), A/Pocheon/SKR/2010 (b), and As1/Shamir/89 (c), and mixture of RNAs transcribed from P1 genes of the three viruses (d)

Multiplex real‐time RT‐PCR was evaluated using the FMD reference strains of seven serotypes and two porcine viruses (SVV and SVDV) inducing FMD‐like diseases. The developed multiplex real‐time RT‐PCR specifically detected and differentiated serotypes O, A, and Asia 1, and did not detect four serotypes (3 SATs and C) and the two porcine viruses (SVV and SVDV) among the reference strains, as shown in Table 2. Standard curves (Figure 1) for the multiplex real‐time RT‐PCR assay were created using transcribed RNA from a plasmid, in which the linearity (*r* ^2^) and efficiency (*E*) were calculated. The *r* ^2^ values were 0.999 for serotype O and Asia 1 and 0.997 for serotype A. The calculated *E* was 101.1, 97.7, and 92.7% for serotypes O, A, and Asia 1, respectively (Figure 1). The LOD of the multiplex real‐time RT‐PCR using the standard RNAs was equivalent to 10, 1, and 1 copies/μL for serotypes O, A, and Asia1 of single or mixed RNA mixtures, respectively (Figure 2). The LOD of the multiplex real‐time RT‐PCR using FMDV serotypes O (O/Anseong/KOR/2019), A (A/Gimpo/KOR/2018), and Asia 1 (As1/Shamir/89) at 10^0^‐ 10^6^ TCID_50_/mL, was 10^1^, 10^1^, and 10^2^ TCID_50_/mL, respectively. Meanwhile, the LOD of the 3D real‐time RT‐PCR was 10^2^, 10^3^, and 10^3^ TCID_50_/mL for FMDV serotypes O, A, and Asia 1, respectively, while that of the VP1 RT‐PCR/sequencing was 10^4^, 10^3^, and 10^2^ TCID_50_/mL, respectively (Table 3).

**TABLE 2:** Serotyping results of multiplex real‐time RT‐PCR for reference strains used in this study

Serotype	Pool region	Topotype/lineage	Strain	GenBank ID	Results
O	1	SEA/Mya‐98	O/Andong/KOR/2010	KC503937	O
O	3	ME‐SA	O/Manisa/Turkey/69	AY593823	O
O	1	ME‐SA/PanAsia	MOG 12/2017	Unregistered	O
O	3	ME‐SA/PanAsia‐2	TUR3/2013	Unregistered	O
O	2	ME‐SA/Ind‐2001d	NEP 33/2017	Unregistered	O
O	1	Cathay	Yunlin/Taiwan/97	KJ831707	O
O	4	EA‐2	KEN 4/2017	Unregistered	O
O	5	WA	GHA 1/2016	Unregistered	O
O	7	Euro‐SA	Campos	AY593819	O
A	1	ASIA/Sea‐97	A/Pocheon/SKR/2010	KC588943	A
A	1	ASIA/Sea‐97	Malaysia97	KJ933864	A
A	3	ASIA/G‐VII	SAU/2/2015	KY982298	A
A	2	ASIA/G‐VII	NEP 12/2017	Unregistered	A
A	3	ASIA/Iran‐05	AFG 50/2017	Unregistered	A
A	3	AFRICA/G‐IV	EGY 19/2016	Unregistered	A
A	3	ASIA/A22	A22 Iraq	AY593762	A
Asia1	1	ASIA/G‐V	MOG/05	EF614458	Asia1
Asia1	1	ASIA/G‐V	CAM/9/80	FJ785228	Asia1
Asia1	3	ASIA/G‐V	As1/Shamir/89	JF739177	Asia1
Asia1	3	ASIA/Sindh‐08	AFG 56/2017	Unregistered	Asia1
C	7	Euro‐SA	C3/Resende/BRA/55	AY593807	‐
SAT 1	6	III	SAT1/BOT/1/68	AY593845	‐
SAT 2	6	II	SAT2/ZIM/5/81	KF112972	‐
SAT 3	6	I	SAT3/ZIM/4/81	KY825732	‐
SVV	‐	Seneca valley virus		KU954086	‐
SVDV	‐	Swine vesicular disease virus		AF268065	‐

**TABLE 3:** Limit of detection (LOD) of the multiplex real‐time RT‐PCR, 3D real‐time RT‐PCR and VP1 RT‐PCR/sequencing for FMDV serotype O (O/Anseong/KOR/2019), A (A/Gimpo/KOR/2018), and Asia 1 (As1/Shamir/89)

	Serotype O			Serotype A			Serotype Asia 1		
	M*	3D**	S***	M	3D	S	M	3D	S
LOD (TCID_50_/mL)	10^1^	10^2^	10^4^	10^1^	10^3^	10^3^	10^2^	10^3^	10^2^

* M, Multiplex real‐time RT‐PCR.

** 3D, 3D real time RT‐PCR.

*** S, VP1 RT‐PCR/sequencing.

### 3.2 Serotyping using field viruses prepared in cell culture

Of the 113 field viruses prepared from LFBK‐α_V_β_6_ cells, 92 and 21 were identified as FMDV serotype O and A, respectively, by the VP1 RT‐PCR/sequencing (Table 4), which were in agreement with the serotypes determined by multiplex real‐time RT‐PCR.

**TABLE 4:** Comparison of FMDV serotyping between multiplex real‐time RT‐PCR and VP1 RT‐PCR/Sequencing for field viruses isolated from FMD endemic countries between 2018 and 2020

Country (Pool region) serotype/topotype/lineage	No. of field viruses*	VP1 RT‐PCR/sequencing	Multiplex real‐time RT‐PCR
Bangladesh (2) O/ME‐SA/Ind‐2001e, n = 5 A/ASIA/G‐VII, n = 7	12	O (5), A (7)	O (5), A (7)
Cambodia (1) O/ME‐SA/PanAsia, n = 33 O/ME‐SA/Ind‐2001e, n = 13 A/ASIA/Sea‐97, n = 12	58	O (46), A (12)	O (46), A (12)
Republic of Korea (1) O/ME‐SA/Ind‐2001e, n = 2 A/ASIA/Sea‐97, n = 1	3	O (2), A (1)	O (2), A (1)
Laos (1) O/ME‐SA/PanAsia, n = 3 O/ME‐SA/Ind‐2001e, n = 11 A/ASIA/Sea‐97, n = 1	15	O (14), A (1)	O (14), A (1)
Viet Nam (1) O/ME‐SA/PanAsia, n = 5 O/ME‐SA/Ind‐2001e, n = 10 O/SEA/Mya98, n = 10	25	O (25)	O (25)
Total	113	O (92), A (21)	O (92), A (21)

* Isolated from LFBK cells.

### 3.3 Comparison of serotyping performance for clinical field samples

To evaluate serotyping performance, serotyping was conducted for clinical field samples (n = 55), and field viruses (n = 45) successfully isolated in cell culture, which was inoculated with the same clinical field samples, using VP1 RT‐PCR/sequencing and multiplex real‐time RT‐PCR. Direct FMDV serotyping rates for field clinical samples were 92.7% (51/55) and 72.7% (40/55) by multiplex real‐time RT‐PCR and VP1 RT‐PCR/sequencing, respectively. The serotyping rate of VP1 RT‐PCR/sequencing was 81.8% (45/55) after viral isolation of field clinical samples in cell culture. Regarding serotyping of VP1 RT‐PCR/sequencing for clinical field samples, serotypes of 40 samples were determined to contain FMDV serotypes O (n = 37) or A (n = 3). In the case of multiplex real‐time RT‐PCR for clinical field samples, 51 samples were determined to support serotype O (n = 43), A (n = 3), or mixed serotypes O/A (n = 5). Mixed serotypes in multiplex real‐time RT‐PCR were confirmed by sequencing the amplicons of the reaction. In serotyping using VP1 RT‐PCR/sequencing after viral isolation, 45 samples were determined to contain serotype O (n = 40), A (n = 3), or mixed serotypes O/A (n = 2) (Table 5). Among the serotypes determined by multiplex real‐time RT‐PCR and VP1 RT‐PCR/sequencing in field clinical samples, the serotype consistency rate between the two assays was 90% (36/40) due to mixed serotypes determined only by the multiplex real‐time RT‐PCR. For the serotypes determined by multiplex real‐time RT‐PCR for field clinical samples and VP1 RT‐PCR/sequencing for viral isolates (after viral isolation), the serotype consistency rate between two assays was 100% (45/45). Meanwhile, among the serotypes determined by VP1 RT‐PCR/sequencing for field clinical samples and viral isolates, the serotype consistency rate was 94.7% (36/38), due to mixed serotypes for two viral isolates.

**TABLE 5:** Comparison of FMDV serotyping of field clinical samples and field viruses obtained from FMD endemic countries (Bangladesh and Cambodia) between multiplex real‐time RT‐PCR and VP1 RT‐PCR/sequencing

					Field clinical sample		Field viruses**
No.	Year	Country	Species	Sample type	VP1 RT‐PCR/sequencing	Multiplex real‐time RT‐PCR	VP1 RT‐PCR/sequencing
1	2020	Bangladesh	cattle	tissues	O	O	O
2	2020	Bangladesh	Cattle	tissues	O	O	O
3	2020	Bangladesh	Cattle	tissues	NS*	O	O
4	2020	Bangladesh	Cattle	tissues	NS	O	O
5	2020	Bangladesh	Cattle	tissues	O	O	O
6	2020	Bangladesh	cattle	tissues	O	O	O
7	2020	Bangladesh	Cattle	tissues	O	O/A	O/A
8	2020	Bangladesh	Cattle	tissues	O	O/A	O/A
9	2020	Bangladesh	Cattle	tissues	NS	NS	O
10	2020	Bangladesh	Cattle	tissues	NS	NS	O
11	2020	Bangladesh	Cattle	tissues	NS	O	O
12	2020	Bangladesh	Cattle	tissues	NS	O	O
13	2020	Bangladesh	Cattle	tissues	O	O	O
14	2020	Bangladesh	Cattle	tissues	O	O	O
15	2020	Bangladesh	Cattle	tissues	NS	O	O
16	2020	Bangladesh	Cattle	tissues	O	O	O
17	2020	Bangladesh	Cattle	tissues	O	O	O
18	2020	Bangladesh	Cattle	tissues	O	O	O
19	2020	Bangladesh	Cattle	tissues	O	O	O
20	2020	Bangladesh	Cattle	tissues	O	O	O
21	2020	Cambodia	Cattle	tissues and saliva	NS	NS	NS
22	2020	Cambodia	Cattle	tissues and saliva	A	A	A
23	2020	Cambodia	Cattle	tissues and saliva	A	A	A
24	2020	Cambodia	cattle	tissues and saliva	A	A	A
25	2020	Cambodia	Cattle	tissues and saliva	NS	O/A	NS
26	2020	Cambodia	Cattle	tissues and saliva	NS	O	NS
27	2020	Cambodia	Cattle	tissues	O	O	O
28	2020	Cambodia	Cattle	tissues	O	O	O
29	2020	Cambodia	Cattle	tissues	O	O	O
30	2020	Cambodia	Cattle	tissues	NS	O	NS
31	2020	Cambodia	buffalo	nasal discharge	O	O	O
32	2020	Cambodia	Cattle	nasal discharge	O	O/A	NS
33	2020	Cambodia	Cattle	saliva	O	O	O
34	2020	Cambodia	Cattle	tissues and saliva	O	O	O
35	2020	Cambodia	Cattle	tissues and saliva	O	O	O
36	2020	Cambodia	Cattle	tissues and saliva	O	O	O
37	2020	Cambodia	Cattle	tissues and saliva	O	O	O
38	2020	Cambodia	Cattle	tissues and saliva	O	O	O
39	2020	Cambodia	Cattle	tissues and saliva	O	O	O
40	2020	Cambodia	Cattle	tissues and saliva	O	O	O
41	2020	Cambodia	Cattle	tissues and saliva	NS	O	NS
42	2020	Cambodia	Cattle	tissues and saliva	O	O	O
43	2020	Cambodia	Cattle	tissues and saliva	O	O/A	NS
44	2020	Cambodia	Cattle	tissues and saliva	O	O	O
45	2020	Cambodia	Cattle	tissues and saliva	O	O	O
46	2020	Cambodia	Cattle	tissues and saliva	O	O	O
47	2020	Cambodia	Cattle	tissues and saliva	O	O	O
48	2020	Cambodia	Cattle	tissues and saliva	O	O	O
49	2020	Cambodia	Cattle	tissues and saliva	O	O	O
50	2020	Cambodia	Cattle	tissues and saliva	O	O	O
51	2020	Cambodia	Cattle	tissues	NS	O	NS
52	2020	Cambodia	Cattle	tissues	O	O	O
53	2020	Cambodia	Cattle	tissues	NS	O	NS
54	2020	Cambodia	Cattle	tissues and saliva	O	O	O
55	2020	Cambodia	Cattle	saliva	NS	NS	NS

* NS, Not serotyped.

** Isolated from the field clinical samples in cell culture.

### 3.4 Detection and serotyping of saliva from FMDV infected pigs

Pigs were challenged with FMDV strains of serotype O, A, and Asia 1, and all of the FMDV‐infected pigs showed typical clinical signs of FMD from 1–3 days‐post challenge (dpc) and viremia (data not shown).

**FIGURE 3:**
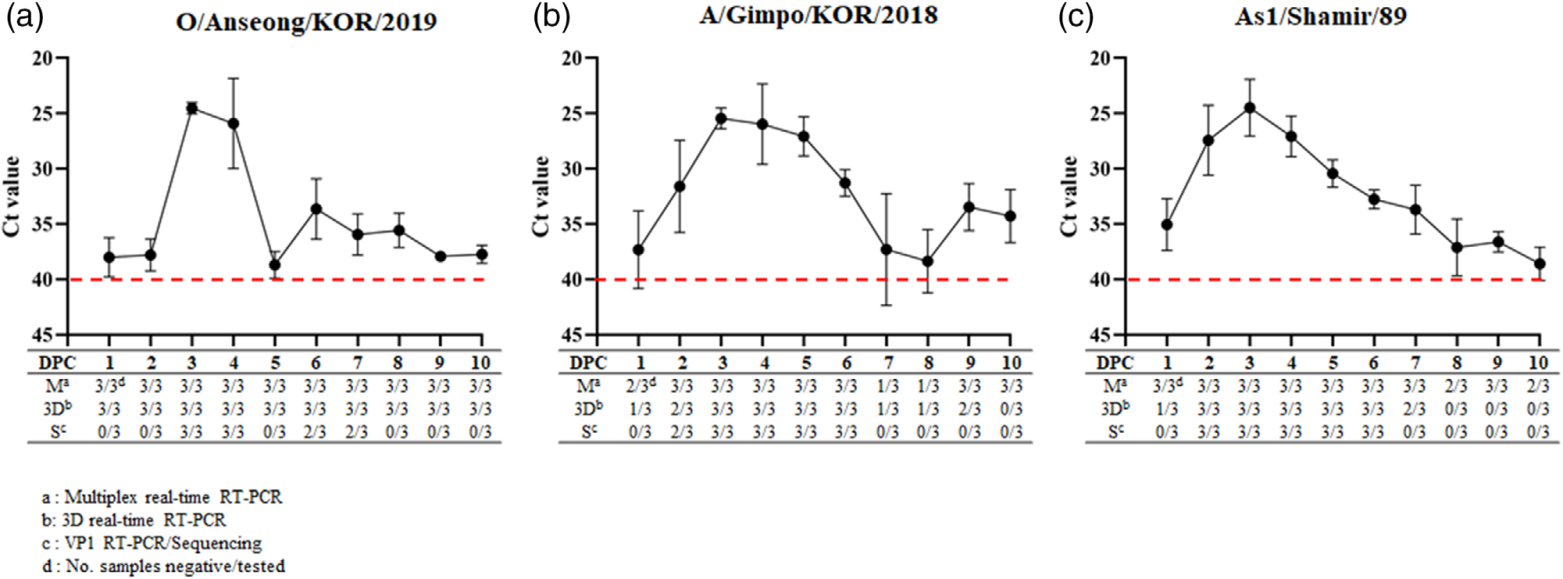
Detection and serotyping of FMDV in saliva collected from experimentally infected pig using multiplex real‐time RT‐PCR for O/Anseong/KOR/2019 (a), A/Gimpo/KOR/2018 (b), and As1/Shamir/89 (c)

In the serotype O infection group, FMD viral RNAs were detected in the saliva of all pigs 1 to 10 dpc, using 3D real‐time RT‐PCR. Saliva was successfully determined as serotype O for all pigs at 3 and 4 dpc, but only two pigs at 6 and 7 dpc using VP1 RT‐PCR/sequencing, while saliva of all pigs was determined as the serotype O using multiplex real‐time RT‐PCR throughout 1 to 10 dpc. In the serotype A infection group, the viral RNAs were detected in the saliva of one pig at 1, 7, and 8 dpc, two pigs at 2 and 9 dpc, and all pigs at 3 to 6 dpc, using 3D real‐time RT‐PCR. Saliva was successfully serotyped as serotype A for two pigs at 2 dpc and all pigs at 3 to 6 dpc using VP1 RT‐PCR/sequencing, while saliva of only one pig at 7 and 8 dpc, two pigs at 1 dpc, and all pigs at 2 to 6 dpc and 9 to 10 dpc was serotyped as serotype A using multiplex real‐time RT‐PCR. In the serotype Asia 1 infection group, the viral RNAs were detected in one pig at 1 dpc, two pigs at 7 dpc, and all pigs at 2 to 6 dpc using 3D real‐time RT‐PCR. Saliva was serotyped as serotype Asia 1 for all pigs at 2 to 6 dpc using VP1 RT‐PCR/sequencing, while saliva of two pigs at 8 and 10 dpc and all pigs at 1 to 7 dpc and 9 dpc was serotyped as serotype Asia 1 using multiplex real‐time RT‐PCR (Figure 3). The *Ct* values of the positive samples ranged from 18 to 44 based on the 3D real‐time RT‐PCR results (data not shown) and from 21 to 39 based on the multiplex real‐time RT‐PCR results (Figure 3).

## 4 DISCUSSION

In the Republic of Korea, a total of 11 FMD outbreaks occurred over 20 years with two serotypes (O and A) of FMDV. These viruses were suspected of being introduced from neighboring countries suffering from FMD endemic with serotype O and A (OIE/FAO Reference Laboratory Network for Foot‐and‐Mouth Disease Annual Reports, 2019). To improve low sensitivity and complicated processes of the current serotyping assays (VP1 RT‐PCR/Sequencing and Ag detection ELISA kit), there have been some attempts to increase serotyping efficiency by developing multiplex RT‐PCR (Alexandersen et al., 2000; Callens & de Clercq 1997; Le et al., 2011; Vangrysperre & de Clercq, 1996) and real‐time RT‐PCR (Jamal & Belsham, 2015; Reid et al., 2014). Nevertheless, these assays are not widely used for serotyping FMDV.

As mentioned earlier, low sensitivity and complicated processes of the current standard assays for serotyping have caused some problems as follows. First, the low sensitivity may lead to failure of serotyping for samples containing low amount of field viruses. There have been reports regarding an increasing rate of untyped FMD suspected clinical samples from 9.2 to 15.2% between 2015 and 2020 (OIE/FAO Reference Laboratory Network for Foot‐and‐Mouth Disease Annual Reports 2015–2020), in which FMDV genomes were positive with RT‐PCR. The failure of serotyping was mainly due to the low sensitivity of the RT‐PCR, which was supported by the result that sequencing following VP1 RT‐PCR had low serotyping sensitivity (10^2^ to 10^4^ TCID_50_/mL) as investigated in this study (Table 3). Besides, there is a low possibility of serotyping all viruses in coinfection with various FMDV serotypes. Coinfection of FMDVs with different serotypes has often been observed in many cases of FMD outbreaks (Al‐Hosary et al., 2019; Gajendragad et al., 1999; Hossen et al., 2014; Maree, 2016; Woodbury, 1994). Second, processes of current serotyping assays, such as VP1 RT‐PCR/sequencing or the antigen detection ELISA, are complicated and time‐consuming. The serotyping of clinical samples by VP1 RT‐PCR/sequencing takes 9–11 h (2–3 h for VP1 RT‐PCR and 7–8 h for sequencing). However, as the sequencing often requires high amounts of amplicons obtained from RT‐PCR, viral isolation and propagation may be first conducted before VP1 RT‐PCR/sequencing, which takes 3–7 days. In addition, antigen detection ELISA requires assay plate preparation, which involves overnight coating of the assay plate with capture antibody and the assay time is typically 4–6 h due to the multiple washing steps and incubation times. The drawback of the current serotyping process may result in serotyping delay and consequent lag in the application of control policies to minimize the FMD spread in emergent situations of FMD outbreaks. These suggest that a novel serotyping method with better efficiency is strongly required to enhance the capability of FMD control.

A multiplex real‐time RT‐PCR assay was developed to detect and differentiate three FMDV serotypes (O, A, and Asia1) directly from clinical samples, improving FMDV serotyping efficiency. The improvement of serotyping performance in the novel multiplex real‐time RT‐PCR was demonstrated at four points. First, the multiplex real‐time RT‐PCR demonstrated the capability to detect and serotype FMDVs from seven FMD pools using reference strains, field isolates, and FMD clinical samples. Also, primers and probes of the assay was designed using VP1 coding regions sequences of O, A, and Asia1 serotypes in seven pools, the assay may have a great potential to be available for FMDVs of all pools. Second, the multiplex real‐time RT‐PCR was determined to be 10 to 100 times and 1 to 1000 times more sensitive than 3D real‐time RT‐PCR for detection and VP1 RT‐PCR/sequencing for serotyping, respectively, depending on the FMDV serotype (Table 3). The enhanced sensitivity in the serotyping was reflected in the improvement in detection capability and serotyping rates of clinical samples from field FMD outbreaks, as well as from pig challenge experiments. For FMD outbreaks clinical samples, the serotyping rate was significantly higher in multiplex real‐time RT‐PCR (92.7%) than VP1 RT‐PCR/sequencing (72.7%) (Table 5). The diagnostic performance for saliva collected from pigs during 10 days after challenge infection with each of the three FMDV serotypes (O, A, and Asia1) was also significantly improved in the multiplex real‐time RT‐PCR. As shown in results, the detection rates of the multiplex real‐time RT‐PCR versus 3D real‐time RT‐PCR were 100% versus 100%, 83.3% versus 63.3%, and 93.3% versus 60% for serotypes O, A, and Asia 1, respectively. More importantly, the serotyping rates of the multiplex real‐time RT‐PCR versus VP1 RT‐PCR/sequencing were 100% versus 33.3%, 83.3% versus 46.6%, and 93.3% versus 50% for serotype O, A, and Asia 1, respectively (Figure 3). Third, the multiplex real‐time RT‐PCR demonstrated the capability to simultaneously differentiate the three FMDV serotypes in a sample containing RNA transcripts (Figure 2) or field viruses (Table 5) of two to three different serotypes, in comparison to VP1 RT‐PCR/sequencing. In particular, as shown in Table 5, five field clinical samples were shown to contain two serotypes A and O by multiplex real‐time RT‐PCR. In contrast, only two of these samples were identified as containing the two serotypes A and O by VP1 RT‐PCR/sequencing using viral isolates, while no sample was identified as mixed serotype in VP1 RT‐PCR/sequencing using clinical samples. Fourth, serotyping using the multiplex real‐time RT‐PCR can be conducted for a high number of samples simultaneously, being less costly and time‐consuming than the current serotyping assays. In addition, since the probes and primers are highly serotype‐specific, serotyping may not be negatively influenced by nonspecific reactions in diverse clinical samples.

Collectively, a novel multiplex real‐time RT‐PCR was developed to detect and differentiate FMDV serotypes using FMD clinical samples in this study. The substantial improvement of diagnostic performance of this new assay was demonstrated for three FMDV serotypes (O, A, and Asia 1) in comparison to the current assays. Nonetheless, the specificity of the novel multiplex real‐time RT‐PCR should be continuously evaluated since the assay targets the most variable region (VP1 coding region sequence) on FMDV genome, which is highly vulnerable to genetic variation by evolutionary pressure (Carrillo et al., 2005). Even more, it would be essential to further evaluate the assay for a practical field application using a large number of FMD clinical samples from endemic regions.

## CONFLICT OF INTEREST

The authors declare that the research was conducted in the absence of any commercial or financial relationships that could be construed as a potential conflict of interest.

## ETHICAL STATEMENTS

The animal experiments were supervised by the Institutional Animal Care and Use Committee of the APQA (IACUC number: 2018–125, 2020–393 and 2021–536) in the Republic of Korea and performed in accordance with the regulations and guidelines of this committee.

## Data Availability

Primer and probe sequences are the primary data. A summary of standard curve and amplification curve data is also included; raw data are available upon request.
